# Machaon, Son of Asclepius, the Father of Surgery

**DOI:** 10.7759/cureus.7038

**Published:** 2020-02-19

**Authors:** Dimitrios Filippou, Gregory Tsoucalas, Eleni Panagouli, Vasilios Thomaidis, Aliki Fiska

**Affiliations:** 1 Surgical Anatomy, School of Medicine, National and Kapodistrian University of Athens, Athens, GRC; 2 Anatomy, Democritus University of Thrace (DUTH), School of Medicine, Alexandroupolis, GRC; 3 Anatomy, National and Kapodistrian University of Athens, Athens, GRC

**Keywords:** trojan war, homer, iliad, battlefield surgery, ancient hellenic mythology

## Abstract

The first description of organized surgical care is given in Homer’s epic poem “Iliad’’, even though evidence of performing surgical operations can be traced back to the history of ancient civilizations. Machaon (ca. 1300 BC), the son of Asclepius, was described as a skillful and confident therapist, whose lineage ensured a unique training. He lived in an era when the reality was shaped by myths, and natural phenomena were ruled by the will of the Olympian gods. It was at that time when philosophers and scientists rediscovered the world that surgery was born. We review and present Machaon’s story, as he is not only the first documented surgeon ever mentioned in written records in Greek history, but he also proved himself to be a valiant soldier at the battlefield, during the Trojan War. It is no wonder that the life of such a charismatic man, living in a place and time of prosperity and steady evolution of both the scientific and the spiritual world, became a demigod patron of surgical treatment and a landmark in the history of medicine.

## Introduction and background

There was a very long period in human history when science, witchcraft, and superstition were closely connected and conflicted. Although the ancient Greeks felt the need to develop ways to treat diseases and heal injuries, their attempts were attached to religion and the supernatural. Magic and medicine were the two sides of the same coin, a process run by ancient priests [1].

During these pre-historical times, several civilizations had developed worldwide. Some of the most important in human history evolved around the Mediterranean basin and in the Middle East: the Egyptians, the Sumerians, the Babylonians, and the Persians; each of them was defined by different social and religious customs. All these archaic cultures possessed and developed knowledge, acquired for practical purposes, with no desire for spiritual or philosophical considerations. They did not try to understand, decode and interpret the very "essence of life" and discover the deeper causation of matter and cognitional beings; therefore, they failed to transform knowledge into wisdom or science [2].

Ancient Greeks were somehow different, as their culture had been inspired by an extroverted and free spirit. They always looked for the cause of things, obvious or not, and never stopped wondering and trying to comprehend the laws of natural world. The Greek intellectual approach to life is summarized in the words of Aristotle (ca 384-322 BC), "all that exists are either known or should be defined" (Greek fragment: εἰ γάρ ἒστιν γνωστόν τό τί ἤν εἶναι, εἰ ὁρίσασθαι). Soon, philosophy was implanted in all disciplines, including medicine, to sterilize it from religious and folklore practices [2]. The critical and philosophical way of thinking facilitated the organization of the existing knowledge and the development of science, which illuminated neighboring civilizations. This sui generis ability has been noted by Plato (ca 448-347 BC) as a particular and unique feature of ancient Greeks, "whatever the Greeks receive from the barbarians, they will eventually work to make it much better", (Greek fragment: ὂτιπερ ἂν Ἓλληνες βαρβάρων παραλάβωσι, κάλλιον τοῡτο εἰς τέλος ἀπεργάζονται) [3].

**Figure 1:**
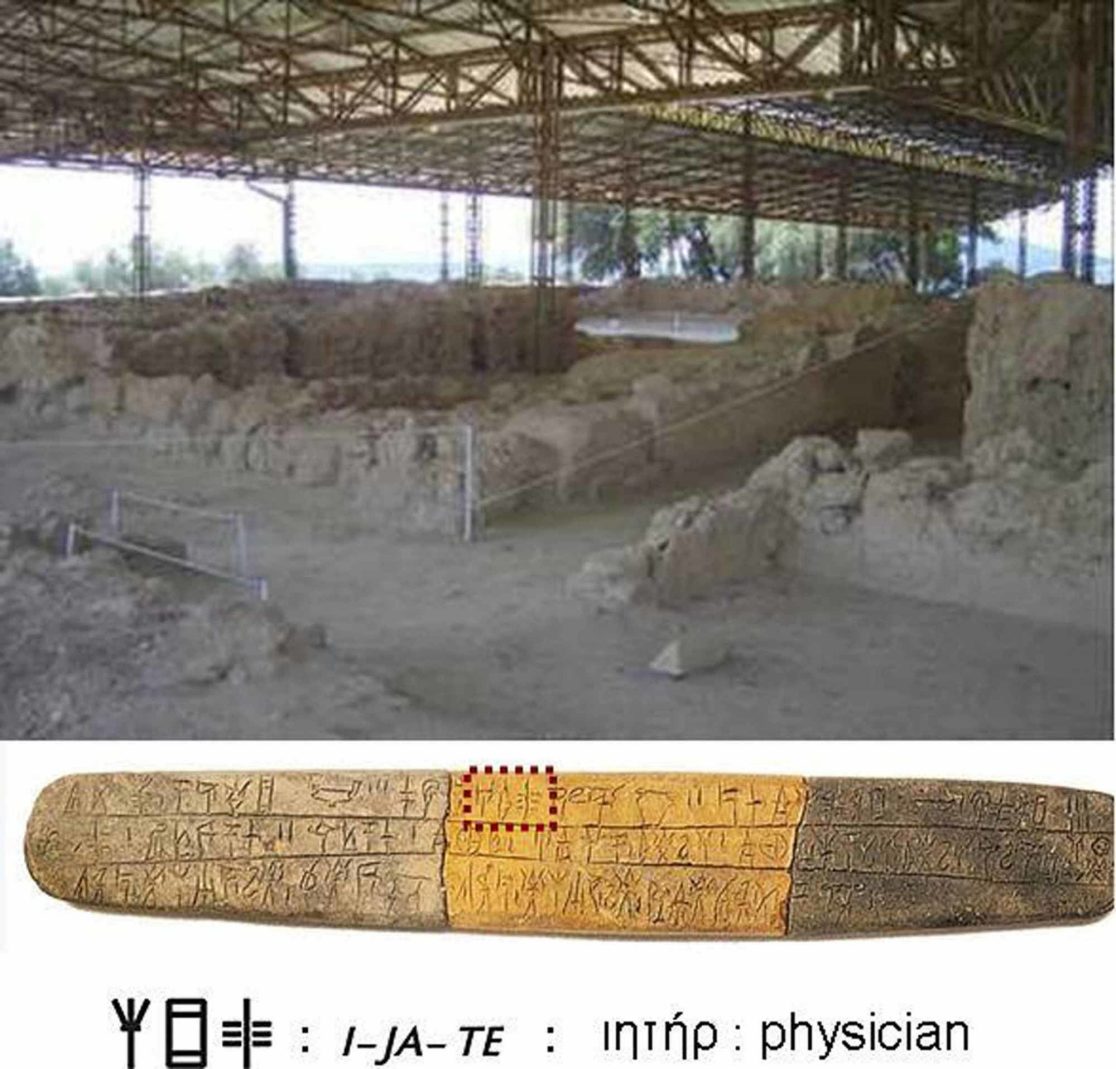
Nestor's palace in Ano Eglianou Nestor's palace in Ano Eglianou, Pylos (top side) and the Mycenaean Table with the term physician engraved in Linear B, ca 2300 BC, National Archaeological Museum of Athens, Eq 146. [Personal collection of G.Tsoucalas]

**Figure 2:**
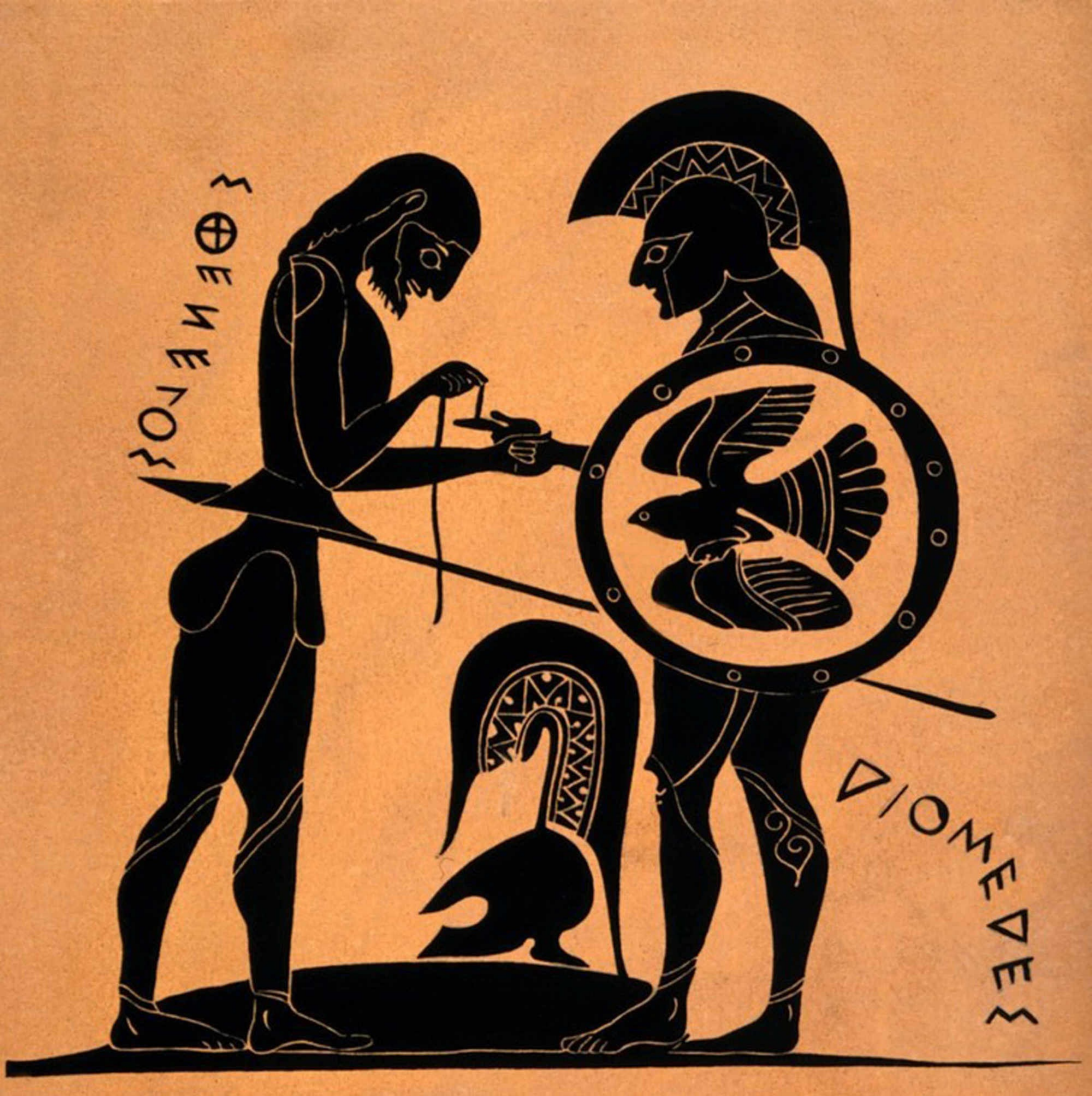
Sthenelos bandaging the wounded finger of Diomedes Sthenelos bandaging the wounded finger of Diomedes, ink drawing after a Chalcidian neck-amphora (now lost) ca 550 BC [https://el.wikipedia.org/wiki/%CE%91%CF%81%CF%87%CE%B5%CE%AF%CE%BF:Sthenelos_bandaging_the_wounded_finger_of_Diomedes._Ink_draw_Wellcome_V0015940.jpg]

Although major developments that changed world history and shaped modern medicine took place in ancient Greece, the lack of written records hampers our knowledge of them. Almost 3000 years ago, the great Trojan War took place: a fierce clash that lasted for 10 years. Many other battles had already taken place in human history, but this was spectacular in numbers and brutality as the ancient Hellenic world collided with Trojans during a long-lasting siege. A detailed description of this war was saved in the epic Homers’ "Iliad" and narrated from mouth to mouth through the ages until it was finally written down in the version available today. A blend of history and mythology created Iliad’s epos, which included the starting point of surgical science and introduced Machaon as the Father of Surgery [4]. Overall, the term "physician" (ancient Greek: ιητήρ) is frequently mentioned in Iliad's text. In addition, a Mycenaean clay Table from "Ano Eglianou Palace" in ancient Pylos, written in Linear B, testifies the existence of physicians in the ancient Hellenic world [Figure 1]. A series of physicians is mentioned by Homer into Iliad epos [Table 1] [Figure 2] [5-6]. The notion that surgical interventions during wars greatly contributed to the development of surgical practice, was summarized in the Hippocratic aphorism "War is the surgeons’ school and everyone who wishes to practice surgery must go to war" [7]. This historical review tries to narrate Machaon’s tale and unearth the relevant scientific data from ancient Greek mythology.

**Table 1:** Physicians mentioned inside the Homeric epos Iliad - The "Ieteres" (ancient Greek: ιητήρες)

Ν	Hero-physician	Patron-Teacher
1	Achilles	Centaur Chiron
2	Machaon	Asclepius
3	Podalirius	Asclepius
4	Patroclus	Achilles
5	Sthenelos	Self-taught

## Review

Divine nature of medicine and surgery

In Greek mythology, disease was Olympian Gods’ retribution for human sins. With the development of organized ancient Greek societies, people’s frustration had a big impact on religion, since punitive Gods were no longer able to meet the needs of the common people. Gods had to acquire blander and more comforting characteristics and as a result, secondary deities came to fill the gap. It was the time for new religious practices to flourish, so that common people’s heroes could rise to divinity. Thus, apart from the Pantheon in Mount Olympus, a new breed of gods arose, amongst which Asclepius. Asclepius was a member of the Argonauts and a skilled war-surgeon who practiced and mastered his techniques in ancient Greece (Greek: την τέχνην ασκήσας επί πολύ και γενόμενος χειρουργικός) [8-9]. Homer suggested that Asclepius was not a god; he was a human hero, with great medical skills. Nonetheless, he was believed to be the son of Apollo and was worshipped as the patron god of Medicine for 2 millennia after his death. More than 320 temples were built to worship, honor, and commemorate him in Magna Graecia [10-11].

**Figure 3:**
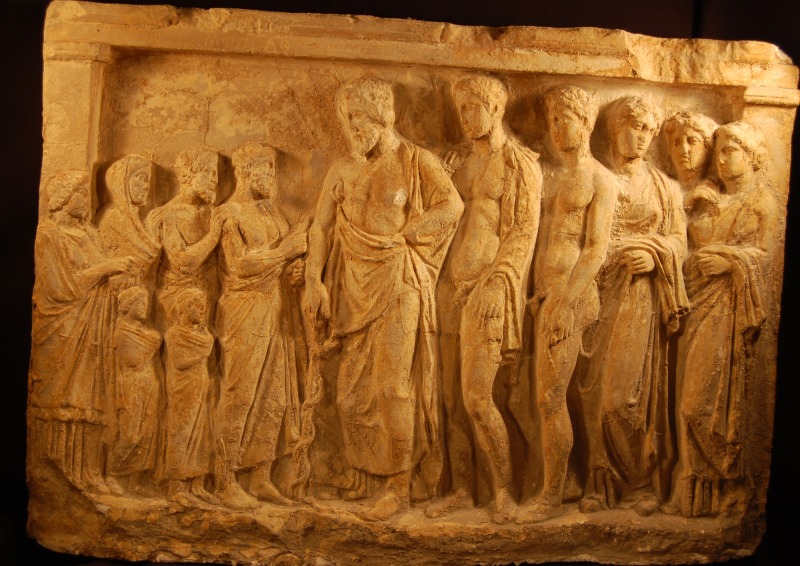
Asclepius with his sons Asclepius with his sons Podalirius and Machaon and his three daughters, with supplicants, Greek relief ca 470-450 BC, found at Thyrea, Greece, National Archaeological Museum of Athens. [https://wellcomecollection.org/works/ekhkh79b]

Asclepius married Epione, who gave him five immortal daughters and two mortal sons. His daughters, Hygieia, Aceso, Iaso, Aegle, and Panacea, represented the stages of the healing process and pharmaceutical treatment. His sons were Machaon and Podalirius, who both became famous physicians [Figure 3] [12]. However, Machaon is mentioned in Iliad’s verses 11 times, while Podalirius only twice [5].

Machaon, the father of surgery

Machaon (Greek: Μαχάων) was the eldest son of Asclepius and the most famous among his children. His name derives from the Greek word "μάχη" (machi), which means battle, or from the word "μάχαιρα" (machaera) which means knife (or scalpel in surgery). Thus, "Machaon" means the man who fights, the warrior and the one who cures with his surgical skills. Machaon studied medicine at Mount Pelion under the guidance of Centaur Chiron who was the esteemed teacher of the first physicians. His tutoring proves the divine origin of Machaon’s medical expertise. Furthermore, the ancient texts suggest that Asclepius himself taught both his sons the basic principles of medicine. Podalirius was the internist, but Machaon became synonymous to traumatology and surgery. The name of Machaon supports the bipolar nature of things in ancient Greece, like war and peace, good and bad, trauma and healing. The soldier injures and kills, while the physician helps and cures. Injury and therapy were the two opposite cornerstones of medicine [1,13,14,15].

According to myth, the Greeks attacked Troy to take back Helen, the wife of Menelaus, King of Sparta, although modern historians suggest that the real motives were mostly economic, commercial and military. King Menelaus and his brother Agamemnon, King of Mycenae and commander-in-chief of all Greeks, asked the two sons of Asclepius to take part in the expedition. Machaon and Podalirius, the commanders of Trikki and Ithome, agreed to join the Greek forces, not only as physicians, but also as the leaders of an army of 30 ships manned with "hoplites" (Greek: οπλίτες, men in arms) [13].

**Figure 4:**
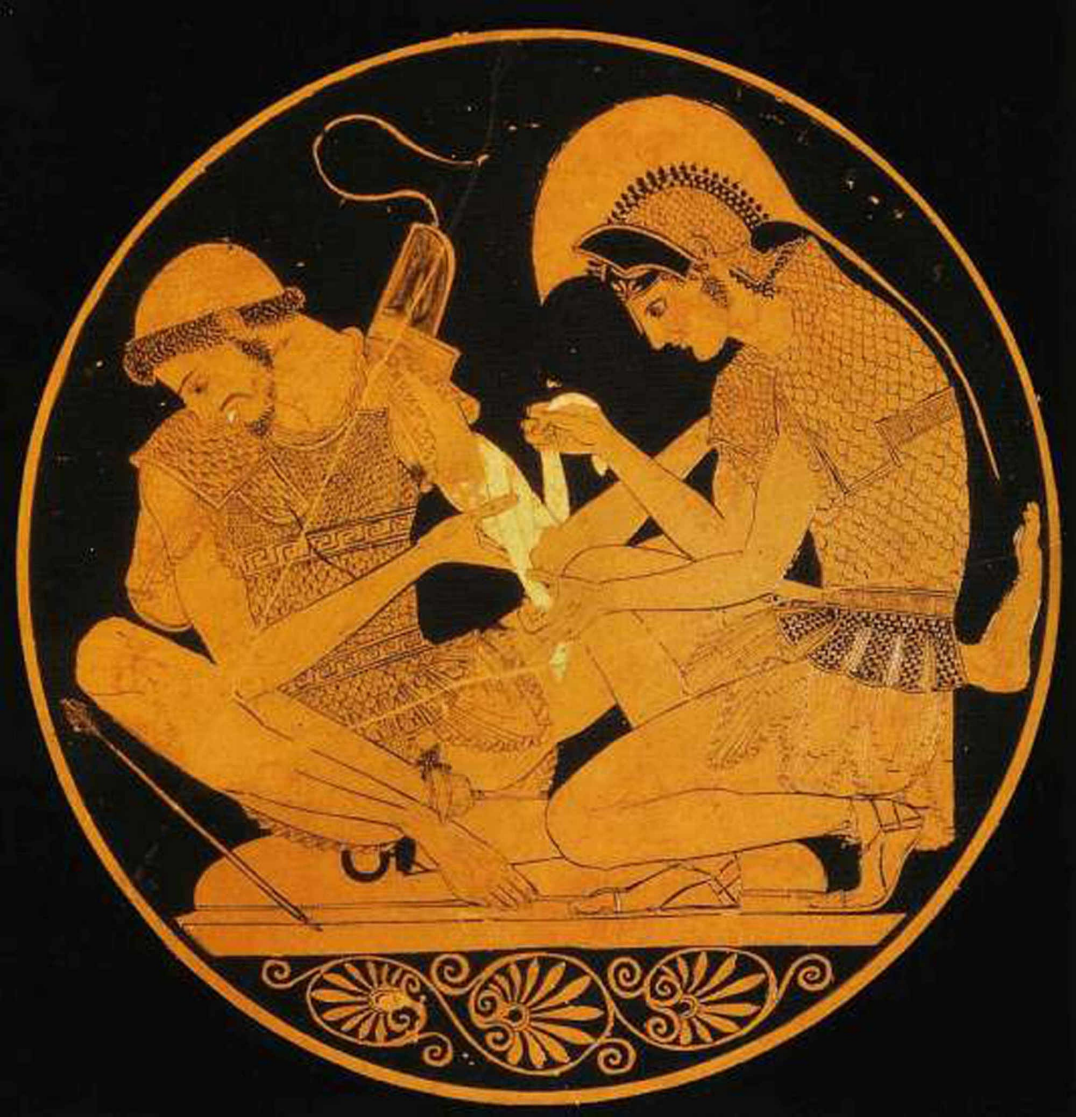
Achilles tending Patroclus Attic red-figure kylix, ca 500 BC, Antikenmuseen, Berlin. [https://commons.wikimedia.org/wiki/File:Akhilleus_Patroklos_Antikensammlung_Berlin_F2278.jpg].

Medical attention of the wounded was well organized in the camp of the Greeks in the coastline outside Troy’s walls. Apart from the rituals of supplications and sacrifices towards gods, daily medical practice included cauterization, herbal medicine, bandaging [Figure 4], wound care and surgery. As a surgeon, Machaon was responsible to take out the arrows and to try to heal all the traumas by using herbs: "Machaon, healer of the traumas, should remove the arrows and treat the wound with mild [herbal] drugs" (Greek fragment: ὃ μέν περί τά τραύματα ἦν (Μαχάων).... τόν Μαχάονα, ὃν μόνον χειρουργεῑν τινές λέγουσιν..... ἰούς τ’ ἐκτάμνειν ἐπί τ’ ἢπια φάρμακα πάσσειν) [16]. Surgery was being practiced purely as a medical art and various primitive surgical tools existed to help with the operations [17].

**Figure 5:**
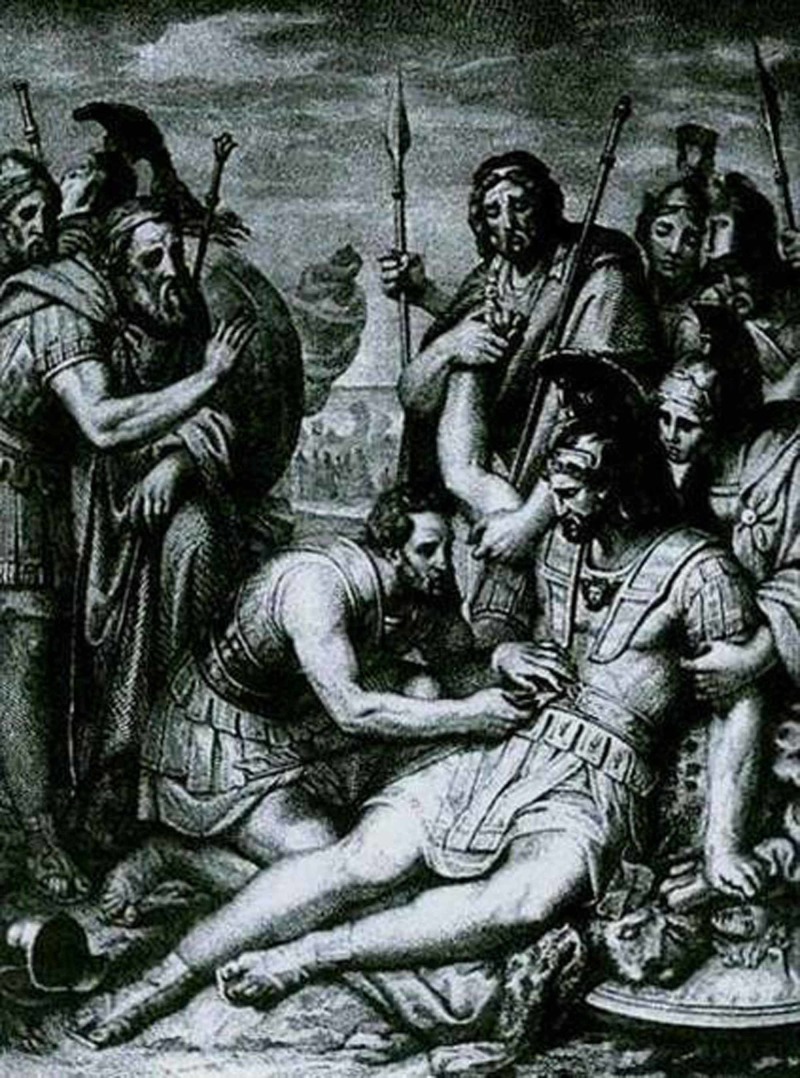
Greek swordsmen observe Machaon Greek swordsmen observe Machaon who removes the arrow from the wound of the chest of Menelaus, chalcography, Nenci F, 1837. [https://classical-inquiries.chs.harvard.edu/a-sampling-of-comments-on-iliad-scroll-4/]

During a ferocious battle, King Menelaus was injured by the arrows of the Trojan aristocrat and celebrated warrior Pandarus. Agamemnon urgently called Machaon to his brother’s rescue. Machaon, successfully removed the broken arrow, aspirated and expectorated the blood from the wound and then applied herbs and drugs (cataplasms, ointments) on the wound in order to control bleeding and promote antisepsis and healing. According to the myth, the medication used for Menelaus' wound was given as a gift from Centaur Chiron to Machaon’s father Asclepius. In the battlefield: "blonde Menelaus laid on the ground and the most glorious kings stood around him. The hero (Machaon) ran between them and approached the injured King. He violently shook the buckle from his tight belt and by pulling it the belt was loosened; he opened last the girdle, bronzed by wondrous smiths. Then he saw the wound, where the arrow was embedded, he extracted it, sucked out the blood and he applied herbal salves on it, which Chiron had kindly given to his father" (Greek fragment: Ὧς φάτο, τῷδ’ ἅρα θυμόν ἐνί στήθεσσιν ὂρινε ... ὄθι ξανθός Μενέλαος βλημένος ἦν ... αὐτίκα δ’ ἐκ ζωστῆρος ἀρηρότος ἒλκεν ὁ ἱστός ... αὐτάρ ἐπεί ἲδεν ἒλκος ὃθ’ ἔμπεσε πικρός ὁ ἱστός ... αἶμ’ ἐκμυζήσας ἐπ’ ἂρ’ ἢπια φάρμακα εἰδώς πάσσε ... τά οἱ ποτέ πατρί φίλα φρονέων πόρε Χείρων). This is the first recorded attempt to heal a wound, the first adequately described intervention in terms of surgery more than 3 millenia ago [Figure 5] [18-20].

**Figure 6:**
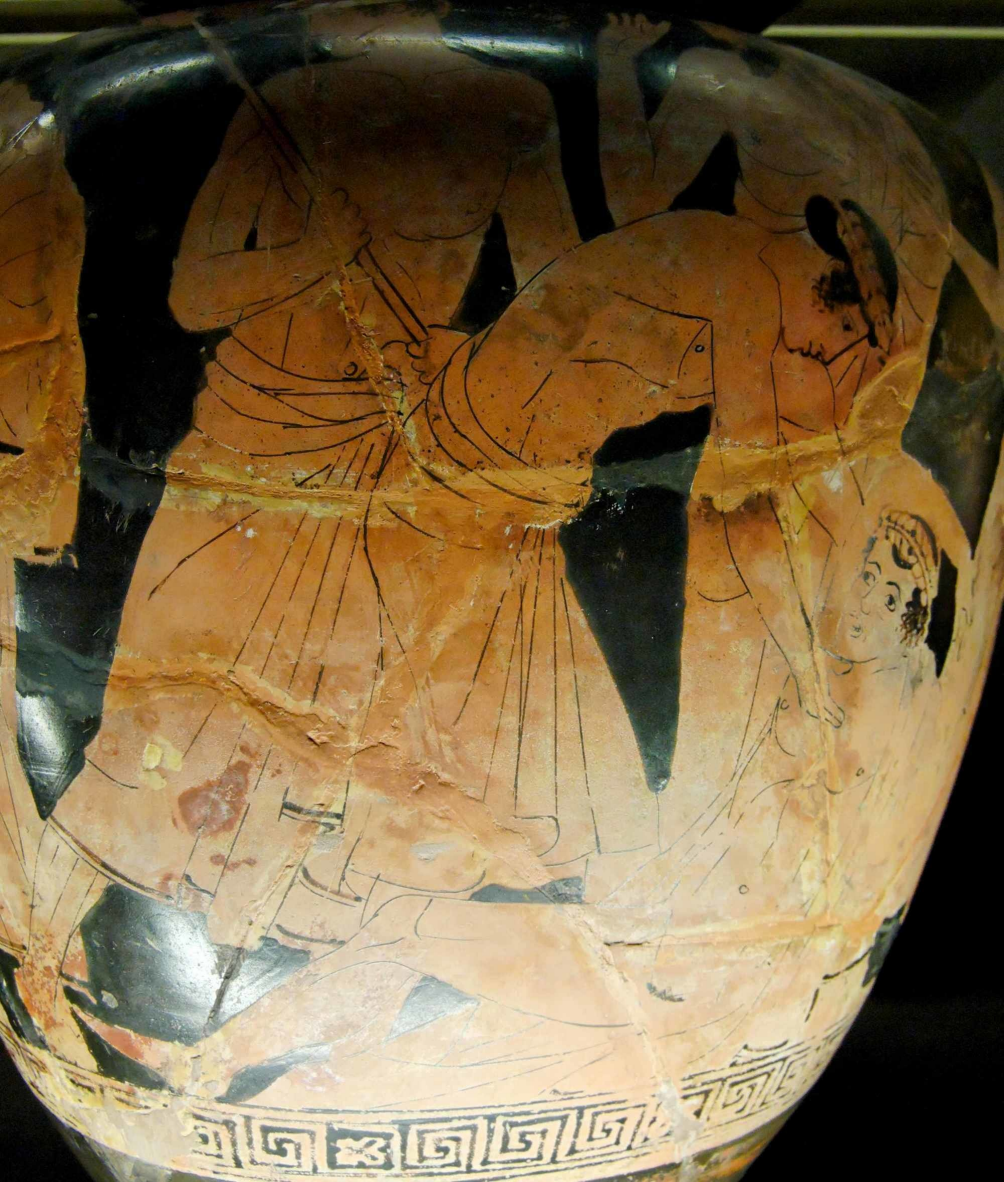
Philoctetes, wounded, is abandoned by the Greek expedition en route to Troy Philoctetes, wounded, is abandoned by the Greek expedition en route to Troy, detail of an Attic red-figure stamnos, ca 460 BC, Musée du Louvre, Paris. [https://en.wikipedia.org/wiki/Philoctetes#/media/File:Philoctetes_Hermonax_Louvre_G413.jpg]

**Figure 7:**
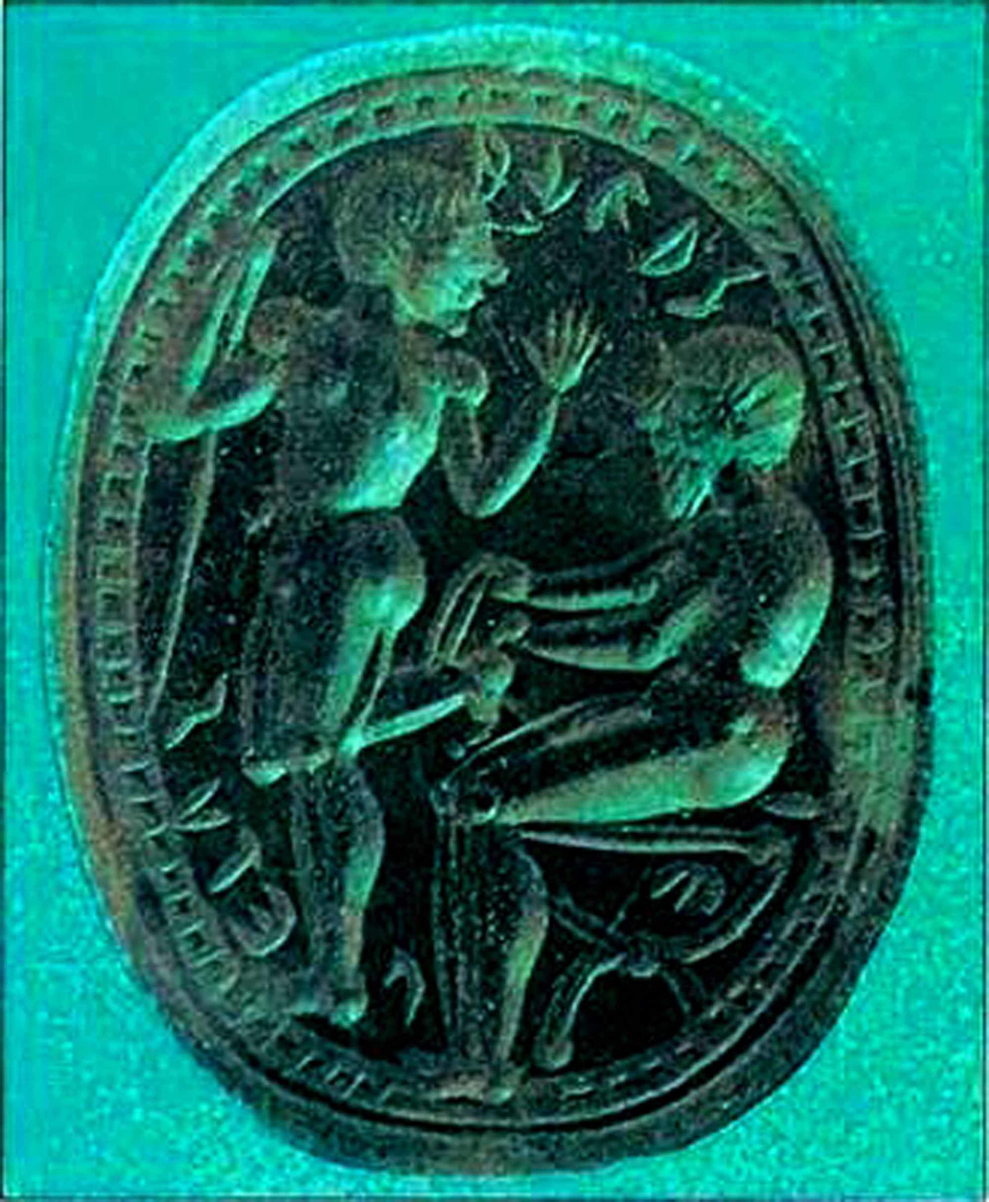
Machaon binds Philoktitos who turns to speak to him Machaon binds Philoktitos who turns to speak to him, Etruscan sealing stone, 2nd half of the 5th century BC, British Museum, London [https://www.quora.com/Can-people-who-understand-Attic-Greek-understand-Koine-Greek-and-vice-versa].

Although he is best known as the healer of Menelaus, this wasn’t Machaon’s only medical achievement mentioned. He had also cured the ancient hero Philoctetes who was the leader of the Thessalian troops in the Trojan War. During a visit in the island of Limnos, Philoctetes was bitten by a snake in his leg. The poison soon led to inflammation and sepsis and his co-warriors had to leave him on the island to die [Figure 6]. At that time the events of war were not favoring the Greek army, so the Achaeans (a term summarizing the Greeks) remembered an old prophecy of the Delphi Oracle which suggested that Troy couldn’t be destroyed without Philoctetes' contribution. They immediately decided to bring him back to Troy, where Machaon healed his septic wound [Figure 7]. Quoting Homer: "Odysseus let him down there, beaten by a snake, […] and Machaon removed the rotten fleshes from the ulcer, putting on it wine and herbs, given to him by Asclepius, gifts of Chiron, and this way he healed the hero" (Greek fragment: ἐκεῑ γάρ αὐτόν ἔρριψεν Ὀδυσσεύς δηχθέντα ὑπό ὃφεος ὒδρου, […] τόν δέ Μαχάονα ἀφέλοντα τοῦ ἒλκους τάς διασαπείσας σάρκας καί ἐπικλύσας οἲνῳ καί θείς βοτάνην, ἢν Ἀσκληπιός εἲληφε παρά Χείρωνος, καί οὒτως ὑγιασθῆναι τόν ἢρωα) [18].

 

During the most notorious war expedition of the ancient world, the ancient Greek aphorism "ἱητρός γάρ ἀνήρ πολλῶν ἀντάξιος ἂλλων", meaning "the physician's value is equal to this of many men" was put in the test and, regarding Machaon, it was proven right. This axiom was firstly said by Idomeneus, the King of Crete and grandson of Minos, referring to Machaon, who cured him after being injured from an arrow outside Troy’s great wall [21].

When Machaon himself was injured by Paris, the Trojan prince, with a three-tongue arrow, all Greek warriors rallied to protect him from being taken or slaughtered by the enemy troops. He was considered so important, that even the great Achilles sent his closest and most trusted co-warrior Patroclus to assess the situation and offer his help before King Nestor of Pylos successfully treated Machaon with warm baths and herbal drugs [5,22-23].

Machaon was not only an excellent healer and surgeon but a brave warrior as well, as he faced the horrifying Evrypylous, Hercules’ nephew, in order to save Nireus and his armor. He participated in the funeral games held in honor of Patroclus, gaining the fourth prize and he was among the brave few who entered Troy inside the Trojan horse [24].

According to the great epic poet, Machaon died in a battle, fighting bravely: "Machaon, the best between all, the leader of the people" (Greek fragment: παῦσεν ἀριστεύοντα Μαχάοντα ποιμένα λαῶν) [18]. Although Machaon was of noble birth and his life in battles enhanced his heroic character, the important position he held in Greek society was mainly based on his role as a surgeon and trauma therapist. His life represents the paradox of war; on one hand he was trying heroically to save the lives of his wounded co-warriors and on the other he was taking the lives of the enemy in battle. He was deified for it [25].

Death and legacy

There is still a great debate around Machaon's death. Appolodorus the Athenian, a great historian who lived in the 1st century BC, in his work "Chronicles" describes the events which took place during the Trojan War. He claims Machaon was killed by the Amazon Penthesilea. Other scholars suggest that Machaon may have been killed by the arrow of Paris during a battle, as it remains unclear if Machaon ever recovered after this injury [26].

Most of the ancient historians, including Quintus Smyrnaeus, and Strabo, support the theory that Machaon was killed by Eurypylus, son of Telephus, who was a formidable warrior. A tremendous battle took place between the two heroes, since Machaon dared to face the beastly warrior in a fair fight, in order to save the lifeless body of Nireus, who had been previously killed by Eurypylus [12,27]. Homer refers to an intriguing dialogue; while Machaon was severely wounded and laid on the ground, Eurypylus ironically said to him that if he was a real physician, he would know how to cure himself and if his father was really a god, he could also save him by giving him nectar and ambrosia in Olympus. To prove his divine lineage, Machaon foretold that Eurypylus will die during the Trojan War and he will never return to his homeland. Soon after Machaon’s last breath, Eurypylus had a monologue stating that he doesn’t care about death, as nobody was born to live forever. Later, as Machaon had foretold, he was killed by Neoptolemus, son of Achilles and never left Troy [28].

Pausanias who lived almost the same period as Appolodorus (2nd century AD), mentioned that the bodies of Nireus and Machaon were claimed and gained by the Achaeans after a hard battle. The dead bodies of the heroes were burned, and their bones buried in a common grave. After Troy’s destruction, wise the Nestor, king of Pylos, collected the bones of Machaon and brought them back to Greece. Pausanias provided evidence that the remains of Machaon were moved to Gerenia [29].

**Figure 8:**
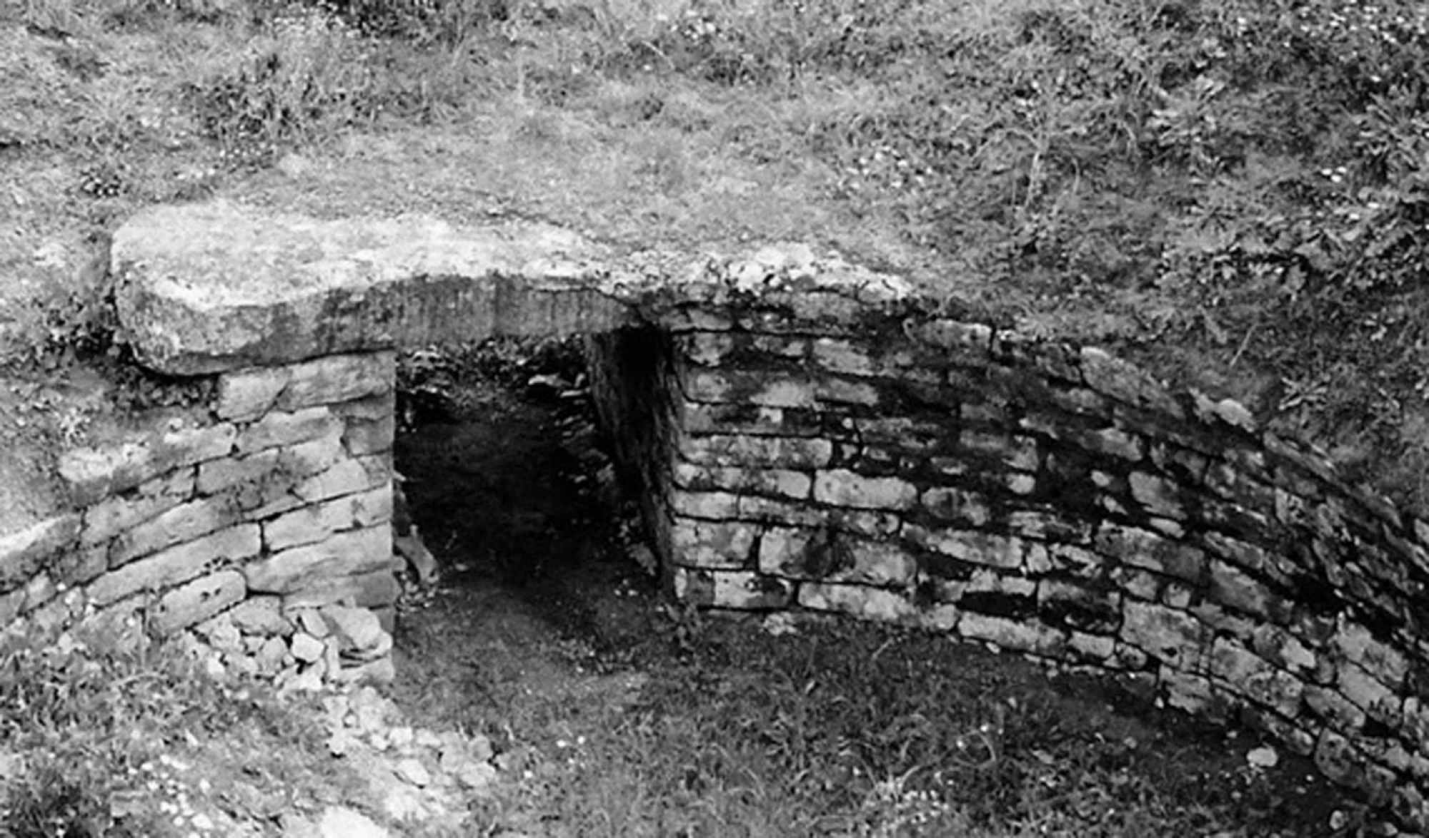
The tomb of Machaon The tomb of Machaon in Necropolis of Gerenia, Messenia, Greece - personal collection of G.Tsoucalas

Many centuries later, in 1891, the excavations brought to light the tomb of Machaon, in the Necropolis of the ancient city of Gerenia, by the archaeologist Christos Tsoundas (1854-1934) [Figure 8]. The large dome of the Mycenaean tomb was well preserved but unfortunately, it had been robbed and only a few burial offerings were found inside the main tomb chamber [30]. The inscription on his gravestone was: "This is the burial place and Shrine of Machaon of Asclepius, a holy place" (Greek: Μαχάονος του Ασκληπιού μνήμα και ιερόν εστίν άγιον). Gerenia is probably the place, where the deification of the mortal heroic warrior and therapist Machaon was initiated soon after his apheroismos (Greek: αφηρωισμός, heroized, distinguished among dead and worshiped as god) [14].

Machaon was married to Anticleia, daughter of Diocles, king of Pherae (Greek: Φαραί) in Messenia. He had six children: Nicomachus, Gorgasus, Alexanor, Polemocrates, Sphyros, and Alcon or Asclepius, according to Democritus from Stagira [29]. After Machaon’s death, his eldest sons, Nicomachus and Gorgasus inherited the Kingdom of Pherae and although they were kings, they had also become physicians, preserving the family tradition. After their death, the people of their kingdom built a sanctuary to honor their memory. As for the other four sons, they also practiced medicine and helped in the construction of sanctuaries, dedicated to their father, the demigod Machaon and their grandfather Asclepius, God of Health [31].

Machaon was worshipped in Asclepieia all over Greece. In Epidaurus, the greatest of all, Machaon was worshipped alongside Asclepius, as his equal, as various ancient inscriptions testify [32].

## Conclusions

Surgery and traumatology were born 3300 years ago in ancient Greece, in an era when heroes and gods, myths, and legends were indivisible. It was the time when all sciences were founded and developed in this small part of the world, the Hellenic peninsula. Machaon, a Greek heroic warrior and king, who ascended to a deity in the ancient world, is deemed as the Father of Surgery. In his name, battle and medicine were attached together and glorified. His divine origin granted prestige to the discipline of surgery, which was celebrated in his name by Homer. Machaon became the first documented surgeon of the history of medicine, opening the way to surgery’s evolution through the eons to come. 

Anatomy and surgery share a common bond, forged in the ancient battlefields. Surgery was born during the ferocious clashes of the antiquity, where warrior heroes had to treat their fellows. Machaon is the first documented surgeon, rejoicing a blended nature of human hero and god physician. In an era of myths and brutality in the Hellenic peninsula, Machaon planted the seed for the most prestigious branch of medicine, and became the father of surgery.
